# Perceived Barriers for Accessing Health Services among Individuals with Disability in Four African Countries

**DOI:** 10.1371/journal.pone.0125915

**Published:** 2015-05-20

**Authors:** Arne H. Eide, Hasheem Mannan, Mustafa Khogali, Gert van Rooy, Leslie Swartz, Alister Munthali, Karl-Gerhard Hem, Malcolm MacLachlan, Karin Dyrstad

**Affiliations:** 1 SINTEF Technology and Society, Oslo, Norway; 2 Trinity College, University of Dublin, Dublin, Ireland; 3 Afhad University for Women, Omdurman, Sudan; 4 University of Namibia, Windhoek, Namibia; 5 Stellenbosch University, Stellenbosch, South Africa; 6 University of Malawi, Zomba, Malawi; University of Perugia, ITALY

## Abstract

There is an increasing awareness among researchers and others that marginalized and vulnerable groups face problems in accessing health care. Access problems in particular in low-income countries may jeopardize the targets set by the United Nations through the Millennium Development Goals. Thus, identifying barriers for individuals with disability in accessing health services is a research priority. The current study aimed at identifying the magnitude of specific barriers, and to estimate the impact of disability on barriers for accessing health care in general. A population based household survey was carried out in Sudan, Namibia, Malawi, and South Africa, including a total of 9307 individuals. The sampling strategy was a two-stage cluster sampling within selected geographical areas in each country. A listing procedure to identify households with disabled members using the Washington Group six screening question was followed by administering household questionnaires in households with and without disabled members, and questionnaires for individuals with and without disability. The study shows that lack of transport, availability of services, inadequate drugs or equipment, and costs, are the four major barriers for access. The study also showed substantial variation in perceived barriers, reflecting largely socio-economic differences between the participating countries. Urbanity, socio-economic status, and severity of activity limitations are important predictors for barriers, while there is no gender difference. It is suggested that education reduces barriers to health services only to the extent that it reduces poverty. Persons with disability face additional and particular barriers to health services. Addressing these barriers requires an approach to health that stresses equity over equality.

## Introduction

Equity in health requires that all individuals and groups have access to health services of good quality, and that services are provided according to individual needs [1]. There is an increasing awareness among researchers and others that marginalized and vulnerable groups may face problems in accessing health care, and that access problems particularly in low-income countries may in fact jeopardize the targets set by United Nations in the form of the Millennium Development Goals [2–4] (MDGs). Based on the World Disability Report [5] (WDR), The World Health Organization (WHO) has lately highlighted access to health services, health care and rehabilitation services as both a human rights issue and a key development issue [6,7]. While some research exists on access to health services for individuals with disability in low-income countries [8], a recent review of research priorities for health of people with disabilities states that identifying barriers for individuals with disabilities in accessing health services should be the leading research priority [9]. An international team with researchers from six different countries recently carried out a comprehensive study in Sudan, Namibia, Malawi and South Africa on access and quality of health services for vulnerable groups. Based on this study, the article presents empirical data on barriers to health services for individuals with disability. The purpose is both to identify the magnitude of specific barriers, and to estimate the impact of disability on barriers for accessing health.

### Access to health services

The World Disability Report [5] (WDR) states clearly that a range of barriers reduces access to health care for individuals with disability. The main empirical basis for this statement is data from the World Health Survey [10] (WHS). WHS includes ten different reasons for lack of care and the presented results from low-income countries indicate that costs related to visiting health care is the most frequent problem. Costs of the visit, inadequate equipment, negative experiences with health care personnel, inadequate skills among health care providers, and direct exclusion (denied care) occur more often as barriers than among non-disabled. WDR further refers to one study in India and three studies in southern Africa that rank costs, lack of services, lack of transportation, and problems with the quality of services as the most important barriers.

In a nation-wide survey in Afghanistan, Trani et al. [11] found that vulnerable groups, including individuals with disability, faced more difficulties while using health centres and hospitals, as well as private providers. At the same time, vulnerable groups reported higher expenditure related to visiting health centers. Cost of care, coverage of remote areas, and transport were identified as main barriers. In a similar study in Sierra Leone, Trani et al. [12] concluded that there was a disparity in access to government-supported health care facilities between persons with and without disabilities, but also that there were no difference in access between women with and without disabilities with regards to reproductive health. The authors further concluded that socio-economic differences were a key factor for explaining variation in access rather than disability itself.

Van Rooy et al. [8] interviewed 25 individuals with disability in rural, northern Namibia and identified lack of transportation, cost of the transportation when available, and availability or distance to care as the main barriers.

While most of the sources referred to in this review seem to converge around the same type of barriers, the research in this area is still limited both in number and geographically. Except for the two studies by Trani et al. [11,12], few, if any, studies have provided an opportunity for analyzing the impact of disability on access to health services when controlling for other well-known phenomena, such as demography and socioeconomic status. The present household survey in Sudan, Malawi, Namibia and South Africa adds to this limited knowledge base.

### Study contexts

EquitAble is a four-year collaborative research project on access to health care for vulnerable people in resource poor settings in Sudan, Namibia, Malawi and South Africa, carried out in 2010–2014. The survey reported in this article was one of three research components in Equitable. The study was carried out in late 2011 and early 2012 in four different sites in each of South Africa, Sudan and Malawi, and five sites in Namibia. The selection of study sites was carried out in each country, with the purpose of including populations with different characteristics, while at the same time highlighting the particular characteristics of each country that had been pre-defined during the development of Equitable; contexts where a large proportion of the population has been internally displaced (IDPs) (Sudan); where the population is highly dispersed (Namibia); where chronic poverty and high disease burden, including HIV, compete for meagre resources (Malawi); and where, despite relative wealth, universal and equitable access to health care is yet to be attained (South Africa). Thus, the selection of sites in each country did not aim for national, representative samples, but to capture specific vulnerable populations in each country.

In Sudan, the four sites were: Umbada locality (western part of Khartoum state), Kassala state (Eastern Sudan), Rabak Locality (Eastern bank of the White Nile), Shikan State (Central part of Sudan). In South Africa, the four sites were: Gugulethu (Western Cape province), Madwaleni (Eastern Cape Province), Worcester (Western Cape province), Fraserburg (Northern Cape province). In Malawi, the four sites were: Blantyre and Phalombe Districts (Southern region), Ntchisi (Central Region), and Rumphi (Northern region). In Namibia, the sites were: Khomas (central region), Hardap (south of the country) Omusati (north), Kunene (northwest), and Caprivi (far north east).

Clusters within all sites were defined by the country teams based on the predefined characteristics as well as practical considerations.

## Methods

The sampling strategy was a two-stage cluster sampling. A flexible approach was applied for the sampling, so that the four Country Teams in dialogue with the Project Leader and the Project Team decided on geographical areas in each country and how to define clusters in the respective contexts. Sample size was set to 400–500 households (HHs) per site in each country. The following sampling strategy was followed in all countries:

1. Definition of clusters within the sites: clearly defined geographical areas (for instance Enumeration Areas, EAs)
2. A listing procedure whereby all HHs in each cluster were screened for disability, using the activity limitation based Washington Group on Disability Statistics 6 questions [13]
3. Random sampling of required number of HHs with at least one disabled member
4. Individual controls were selected within identified HHs, matched by age and gender

In addition to the screening instrument (Washington Group 6 questions), three different questionnaires were applied: a) Household questionnaire mapping a series of indicators on living conditions at the household level, b) Individual questionnaire applied to the identified individual with disability (in some cases more than one person), c) Control questionnaire to a matched (age, gender) individual within the same household. The questionnaires were all based on previous experience with large scale studies of living conditions among people with disabilities in southern Africa [14] and adapted to the particular purpose of the study.

In addition to descriptive statistics and simple statistical tests, we develop a structural equation model (SEM), which serves to estimate the relationship between barriers to health care, disability, and socioeconomic status while controlling for gender, level of education, and urban residence. The model was estimated for the combined sample as well as the respective country samples. A main advantage of SEM is that it can accommodate observed as well as unobserved (latent) variables and fit several regression equations into one model [15]. The models were estimated using the *sem* procedure in Stata 13, with maximum likelihood as estimation method.

### Ethics statement

Ethical clearance was obtained from the responsible authority in each of the participating countries; The Research and Ethical Committee, Afhad University, and The National Scientific and Research Committee, Federal Ministry of Health (Sudan), Health Research Ethics Committee, Stellenbosch University (South Africa), Office of the Permanent Secretary, Ministry of Health and Social Services (Namibia), the National Health Sciences Research Committee (Malawi), as well as the Norwegian Social Science Data Services (NSD). Key ethical issues were included in the survey manual and in the training preceding data collection. A standard introduction was read to all respondents. Participation was voluntary and all participants consented orally. The choice of consent procedure was discussed in the Research Team during the design phase and oral consent was chosen due to the low rates of functional literacy in large parts of the sample. Consent from persons with disability was witnessed by the head of the household or the main care taker. As the instructions read out to the respondents prior to each interview clearly stated that participation was voluntary, responding to the questionnaire was recorded as consent. Training of enumerators was particularly thorough on ethical issues, including procedures for obtaining consent. All enumerators were bound by professional confidentiality, and the data files were anonymized.

## Results

### Sample characteristics

The data used for the present analysis combines household and individual level data. The combined file comprises a total of 9∙307 individuals. A small number of missing values, particularly in Sudan, leads to minor variations in N between different variables. <Table 1 presents summary statistics for the full sample as well as the country subsamples. The variables included in the table are described below.

**Table 1:** Sample characteristics, by country.

Variable		Total	South Africa	Namibia	Malawi	Sudan	Min	Max
Male		0∙39	0∙30	0∙41	0∙45	0∙41	0	1
Age (years)		36∙50 (20∙91)	41∙99 (18∙07)	43∙21 (23∙36)	27∙94 (18∙57)	39∙30 (21∙54)	1	100
Urban		0∙28	0∙47	0∙48	0∙02	0∙35	0	1
Education								
	No formal education	0∙15	0∙17	0∙24	0∙12	0∙05	0	1
	Less than primary school	0∙13	0∙18	0∙27	0∙06	0∙07	0	1
	Completed primary school	0∙45	0∙32	0∙31	0∙65	0∙40	0	1
	Secondary school	0∙14	0∙19	0∙12	0∙11	0∙11	0	1
	Tertiary school	0∙02	0∙04	0∙04	0∙04	0∙004	0	1
Possession scale		0∙19 (0∙18)	0∙30 (0∙20)	0∙25 (0∙19)	0∙09 (0∙08)	0∙19 (0∙13)	0	0∙89
Activity limitation scale		1∙23 (0∙37)	1∙20 (0∙33)	1∙33 (0∙41)	1∙13 (0∙22)	1∙48 (0∙56)	1	4
Barriers to health services scale		1∙58 (0∙71)	1∙45 (0∙76)	1∙45 (0∙49)	1∙59 (0∙52)	2∙11 (1∙06)	1	5
*N*		*9∙307*	*2∙824*	*1∙624*	*1∙526*	*1∙333*		

*Note*: Mean values with standard deviation in brackets (continuous variables only). For dichotomous variables, the mean value represents the share of sample which takes the value of 1.

*Note*: With the exception of the variable Urban in the Namibian sample, all country level differences are statistically significant on a. 01 level or lower.

The gender balance varied significantly between the four country samples. Some of these differences, and the particularly skewed gender balance in SA, are assumed to be due to characteristics of the selected sites, with a high proportion of migrating workforce. Age did also vary significantly between the countries, and between men and women. Mean age for men and women in the total sample was 34∙7 and 37∙9 years respectively, and the gender difference in age varied somewhat between the four countries from 3∙8 years higher mean age among females in South Africa, 1∙5 years higher among females in Malawi, and 1 year higher among males in Namibia and Sudan.

In South Africa and Namibia, the urban/rural distribution is around 45/55, and 35/65 in Sudan, while almost the entire sample (97∙4%) in Malawi is rural, yielding an overall rural proportion of 71∙4 per cent. This was to be expected from the settlement patterns in the respective countries.

Level of education was measured on a five point scale stating the highest level achieved. Some variation was found between the countries, with South Africa scoring significantly higher than the other countries. It is assumed that some of the country differences in reported level of education may be due to difference in mean age between the countries. No gender difference in level of education was found in the overall sample, or in any of the country subsamples. Missing values of education were imputed with the country mean value in the statistical analyses.

As a proxy for socioeconomic status (SES), a Possession scale was constructed on the basis of household ownership (yes/no) of 28 items that were found relevant for the present contexts. The selection of items was based on experiences from previous studies in the region, and was also an outcome of lengthy discussions in the project team. The following items were included: radio, refrigerator, hi-fi/music stereo, microwave, internet access in the home, electricity, DVD/VHS player, solar energy system, cell phone/mobile, electrical generator, telephone in the house, personal computer, iron, bicycle, fan, motorcycle/quad bike, heater, dishwasher, air conditioner, bed(s), stove with gas/electric, livestock (cattle etc.), stove with paraffin, washing machine, sofa, satellite dish, television, and car. A small number of missing values (<50 for each item) were imputed with the mean value of 0. Exploratory factor analysis yielded a Cronbach's α = ∙89, and KMO = ∙94. The items were added together and divided by the number of items, to form a Possession scale ranging from 0–0∙89, where a score of 0 means that the respondent has none of the mentioned items, while. 89 means that the respondent has 89% of the items (mean: 0∙20, st. dev: 0∙18). Substantial and statistically significant variation was found between countries, confirming well-known socio-economic differences between the countries [16] (SA: 0∙30, Namibia: 0∙25, Malawi: 0∙09, Sudan: 0∙19).

Activity limitation was measured by the 6 screening questions developed by Washington Group on Disability Statistics [17], and recommended by WHO^5^ and the UN Statistical Commission for household surveys [18]. Respondents were asked to rate the difficulty they had in seeing, hearing, walking or climbing steps, remembering or concentrating, self-care, and communication, on a scale with the following answer categories: no difficulty (1), some difficulty (2) a lot of difficulty (3), and unable to do (4). Cronbach's α for the items combined was 0∙66, and KMO = 0∙71. The six items were added together and formed an Activity limitation scale with range from 1 to 4, where a score of 4 means that the respondent is unable to perform any of the activities. Mean activity limitations varied significantly between the country samples, with Malawi producing the highest mean and South Africa the lowest (Table 1).

### Problems of accessing health care services

**Fig 1:**
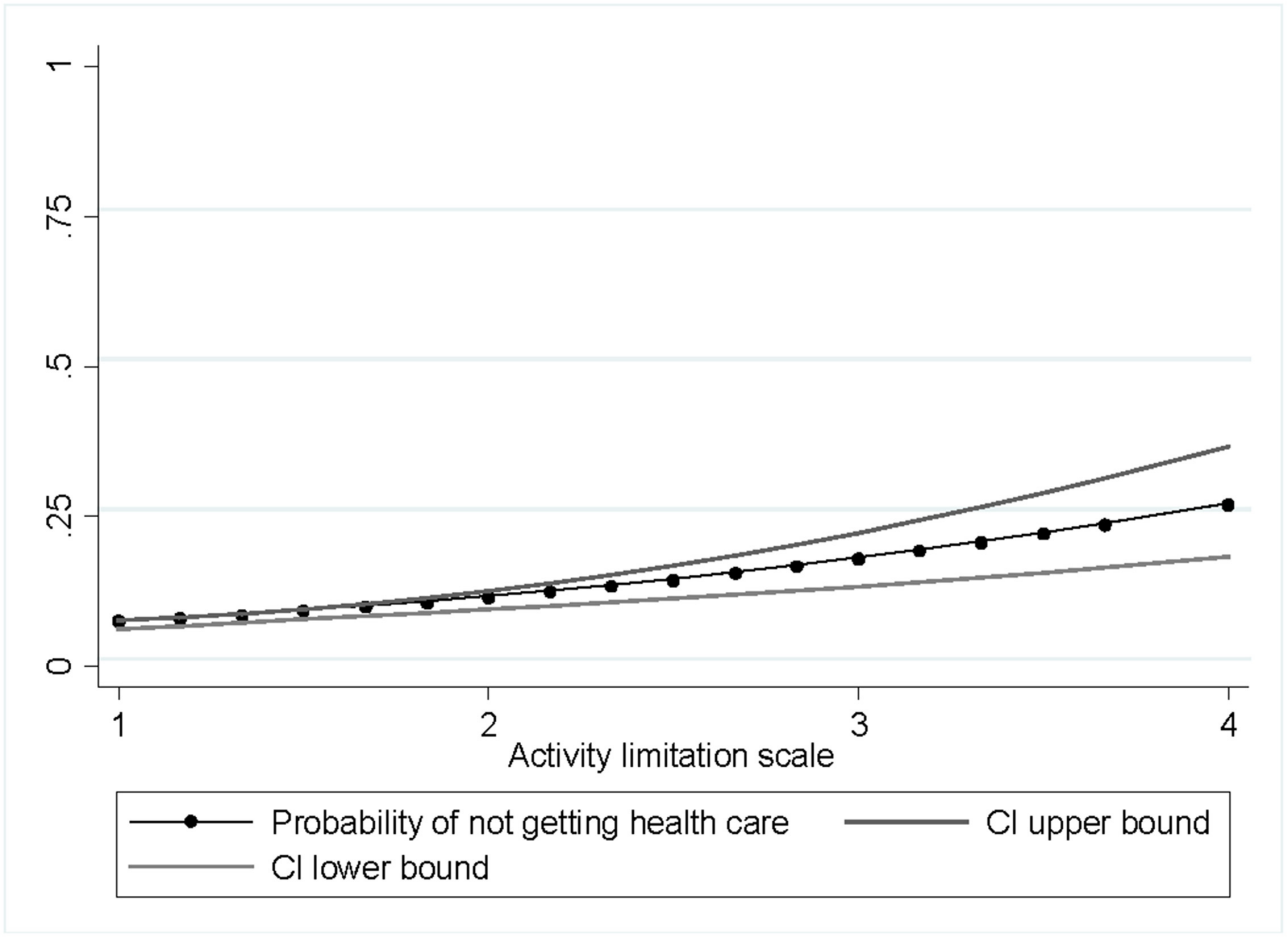
Probability of receiving health care last time needed, with 95 percent confidence interval.

Our data indicate that the probability of not receiving necessary health care increases with level of activity limitation (Fig 1). The question formulation was: "The last time you needed health care, did you get health care?" (yes/no). While the probability of not having received necessary health care was 0∙07 for individuals who reported of no activity limitation, the probability of not receiving such care was 0∙19 for individuals who had severe activity limitations.

Similarly, a much larger share among those with some level of activity limitation reported that availability of health care services had been a problem the last year, as shown in Table 2. The formulation of the question was: "In the past 12 months, how often has the availability of health care services and medical care been a problem for you?" In the total sample, only four percent reported of daily problems with availability. Among those with severe activity limitations, the same share was almost ten percent.

**Table 2:** Availability of health care services a problem, by activity limitation (Percent).

How often has the availability of health care services and medical care been a problem for you (past 12 months)?	Total	No activity limitation	Some activity limitation	Severe activity limitation
Daily	3∙95	2∙51	4∙87	9∙79
Weekly	3∙50	2∙00	4∙73	6∙94
Monthly	10∙10	8∙18	12∙24	12∙10
Less than monthly	11∙98	11∙35	13∙44	7∙65
Never	68∙70	74∙86	63∙30	53∙91
N/A	1∙78	1∙11	1∙42	9∙61
N	9∙172	4∙697	3∙823	562

Note: The difference in availability of health care as a function of activity limitation is statistically significant (p<.001), both in the overall sample and the country subsamples.

### Barriers to access to health care

For a range of items, respondents were asked "Considering your own experience, tell me whether the following make it difficult for you to get health care", with answer categories being "No problem" (1), "Small problem" (2), "Moderate problem" (3), "Serious problem" (4), and "Insurmountable problem" (5). <Table 2 shows the proportion of the sample who had "serious" or "insurmountable" problems in accessing health care.

**Fig 2:**
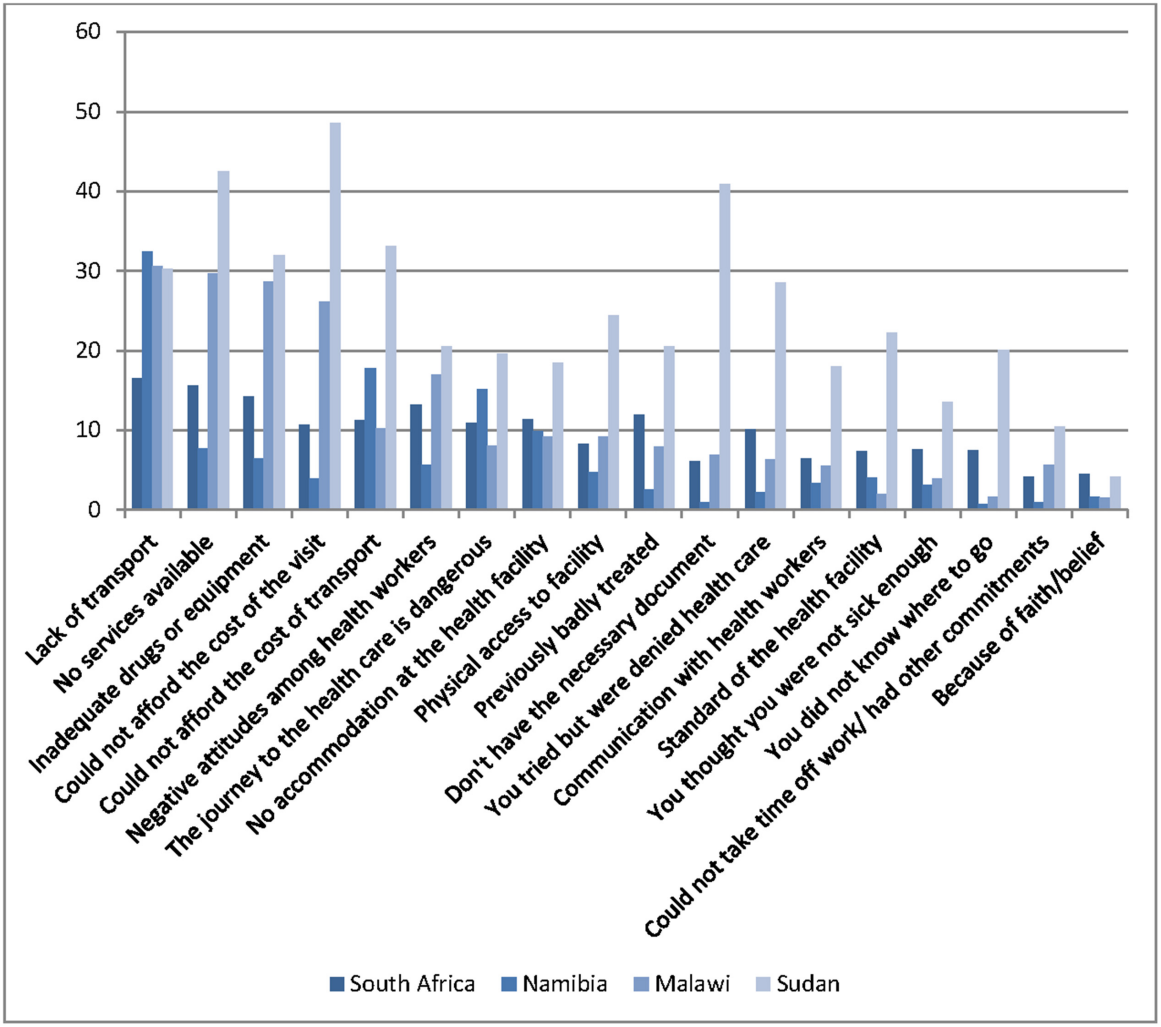
Serious or insurmountable problems in accessing health care, by country. Percent.

The 18 items in <Fig 2 are ranked according to mean values across the four countries. Thus, lack of transport, no services available, inadequate drugs or equipment, and cost of the visit are the four major barriers as perceived by the respondents. The four barriers scoring lowest are faith/belief, lack of time due to other commitments, did not know where to go, and not sick enough. With two exceptions (lack of transport and faith/belief), respondents in Sudan have substantially higher scores than respondents from the other countries. The differences between countries are also statistically significant. The variation in barriers is also higher in Sudan than in the other countries (Table 1). Overall, respondents from South Africa and Namibia reported the lowest barriers. Exploratory factor analysis of the 18 items in <Fig 2 yielded a Cronbach's α of ∙92 and a KMO of ∙95, and supported a one component solution (scree plot, Eigenvalue >1).

**Fig 3:**
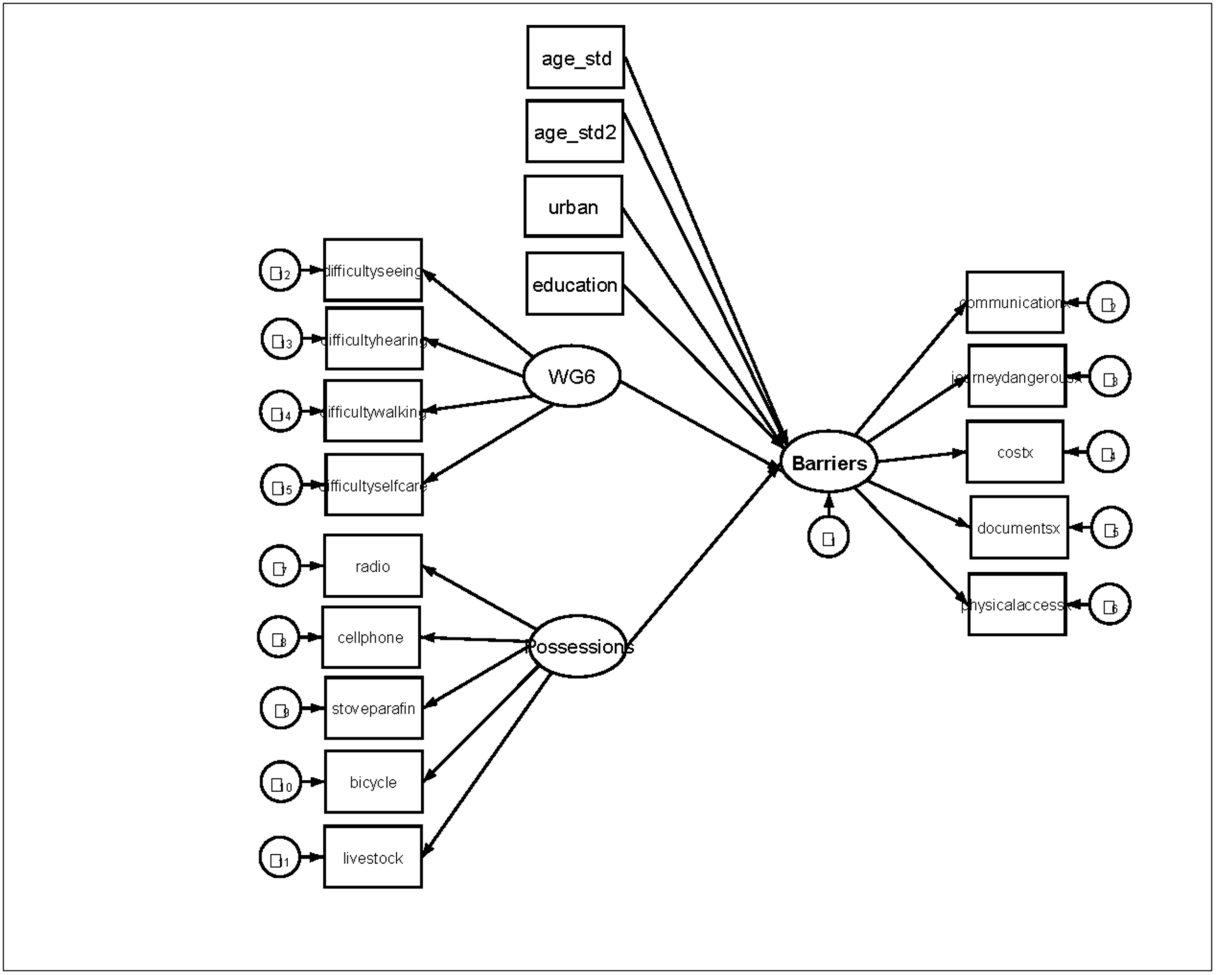
Structural equation model.

To analyze the effects of impairment, socioeconomic status, and other socio-demographic variables on barriers to health services, we estimated a structural equation model (SEM), where socio-economic status, activity limitations, and barriers were treated as latent, or unobserved, variables, which affect individual responses to the possession items, the activity limitation items, and the barrier items, respectively [19]. One possession item, "internet", was excluded from the model due to perfect correlation with another item, "heater", in Malawi. Gender, age, age squared, education, and urban location were measured as exogenous, observed variables. Thus, our model included three measurement models and one structural model, as illustrated in <Fig 3. For simplicity, some of the Possession items and Barrier items are excluded from the figure.

To account for differences between countries, we estimated two different models: one with country dummy variables included as exogenous variables, with South Africa as reference (model 1), and one model where coefficients in the structural model were allowed to vary between countries, while path coefficients in the measurement models were constrained to be equal (model 2). 678 observations were deleted due to missing data on one or more items (listwise deletion). <Table 3 reports the results for the structural model for the two specifications. Output with loadings from the measurement models is not shown, but path coefficients for the items were statistically significant for all three latent variables, for all the items (p<0∙001).

**Table 3:** SEM structural model of barriers to health services, overall and by country.

Variable		Overall	South Africa	Namibia	Malawi	Sudan
Age (centered around mean)		0∙002	0∙005	0∙001	0∙002	-0∙004
		(3∙70)***	(4∙21)***	(-1∙12)	(3∙42)***	(-1∙93)*
Age (centered), squared		-0∙000	-0∙000	-0∙000	-0∙000	-0∙000
		(-5∙72)***	(4∙82)***	(-0∙28)	(2∙40)**	(2∙38)**
Urban		0∙410	0∙811	0∙046	0∙261	0∙142
		(16∙86)***	(21∙07)***	(-1∙66)*	(4∙52)***	(-1∙72)*
Education		0∙018	-0∙031	0∙009	-0∙005	0∙136
		(1∙84)*	(2∙31)**	-0∙83	-0∙46	(4∙33)***
Possessions (latent)		-1∙803	-2∙619	-1∙116	-2∙266	-3∙108
		(-14∙51)***	(13∙29)***	(8∙29)***	(7∙37)***	(4∙66)***
Activity limitation (latent)		0∙506	0∙087	0∙281	0∙576	0∙544
		(8∙86)***	(-0∙91)	(4∙53)***	(5∙.04)***	(4∙95)***
Country dummy variables						
	Namibia	-0∙142	-	-	-	-
		(-5∙88)***	-	-	-	-
	Malawi	-0∙002	-	-	-	-
		(-0∙13)	-	-	-	-
	Sudan	0∙452	-	-	-	-
		(14∙51)***	-	-	-	-
Log likelihood		-388.322∙93	-289.772∙16			
χ^2^ model vs saturated		53.413∙41***	88.422∙80***			
χ^2^ saturated vs baseline		203.314∙76***	178.577∙20***			
AIC		776.999∙86	580.304∙32			
BIC		778.249∙99	582.988∙21			
CFI		0∙74	0∙52			
TLI		0∙73	0∙56			
RMSEA		0∙06	0∙08			
SRMR		0∙06	0∙10			
CD		0∙99	0∙94			
N		8.629	2.652	1.483	3.508	986

*Notes*: z values in parenthesis.

* p<0∙10,

** p<0∙05;

*** p<0∙01. AIC: Akaike's information criterion, BIC: Bayesian information criterion, CFI: Comparative fit index, TLI: Tucker-Lewis index, RMSEA: root mean squared error of approximation, SRMR: Standardized root mean squared residual, CD: coefficient of determination.

<Table 3 confirms some of the findings from the more descriptive analyses, and reveals some interesting effects. As seen in <Fig 2, respondents in Sudan report significantly higher barriers to health services. Age has a positive, statistically significant effect on barriers; i.e., barriers increase with age. The negative, statistically significant coefficient of the squared term indicates that the effect diminishes with increasing age. Urban dwellers face higher barriers than rural, with the exception of Namibia, where the difference between urban and rural dwellers was not statistically significant. Education appears to have an effect on perceived barriers, but the effect differs between countries: In Namibia and Malawi, education has no effect on perception of barriers, whereas barriers decrease with higher education in South Africa, and increase in Sudan. Turning to the other measure of socio-economic status, the latent possession construct, both models show that barriers to health care are reduced with a better socioeconomic position. This effect is consistently strong and statistically significant across model specifications and samples. Finally, disability, measured through the latent activity limitation construct, increases barriers to access to health service in three of the four countries.

### Model fit and robustness of results

There is considerable debate regarding the correct use and interpretation of fitness statistics for SEM [19,20], and several measures of fit statistics are sensitive both to sample size and model complexity. The simple Chi-square test of the discrepancy between the baseline and the model covariance matrices is generally considered a poor measure of fit for larger samples. Our sample is large, and it is not surprising that both models are rejected. Overall, model 1 seems to fare better than model 2, even if none of the models represents a very good fit. The standardized root mean square residual (SRMSR) is lower for model 1 than model 2. A value of 0∙08 or smaller is usually considered a good fit [21]. Similarly, the root mean square of approximation (RMSEA), which accounts for parsimony, is lower for model 1. Here, values of 0∙05 or lower indicate a close approximation, while values up to 0∙08 represent a decent fit. Similarly, the coefficient of determination (CD), which is analogous to R square, is higher for model 1. The Comparative Fitness Index (CFI) and the Tucker-Lewis index (TLI) suggest a poor fit for both models, although somewhat higher for model 1. Given the complexity of the model (i.e., the number of parameters to be estimated), the large sample, as well as the inherent pitfalls of model modifications without theoretical justification, we contend that while not a particularly close fit, the estimated model provides a decent representation of the data. Moreover, differences between the four country samples also imply that it is difficult to find a model that yields a close fit for the whole sample. Looking at the lower-level parameters estimated in the models (e.g., path coefficients), they make theoretically good sense, and none of the parameters takes on unrealistic values. As a robustness check, we estimated the model separately for each country, as well as with a standard ordinary least square regression model with scales instead of latent constructs. These alternative specifications largely confirmed the substantial findings reported in <Table 3, and show that the results are robust to different model specifications and estimation techniques. Gender was excluded from the final models, since initial analyses did not indicate any significant differences between men and women in any of the countries. An alternative model specification which assumed a causal relationship between activity limitations and socioeconomic status produced a clearly poorer model fit.

## Discussion

The above analyses stem from a unique multi-country study on accessibility and quality of health services for individuals with disabilities in four sub-Saharan countries. The study shows that lack of transport, availability of services, inadequate drugs or equipment, and costs are the four major barriers for access. The study also shows substantial variation in perceived barriers between the four countries, largely reflecting the socioeconomic status of the respective contexts. Urbanity, socio-economic status, and severity of activity limitations are important predictors for perceived barriers, while there is no difference between the access of men and women. Education has a more mixed role. Education appears to reduce barriers in South Africa only. One interpretation of this could be that education reduces barriers to health services only to the extent that it reduces poverty. This interpretation finds some support in the data, in particular in South Africa and Namibia, where the inclusion of a path between education and SES indicates that education significantly increases SES, which in turn reduces the perceived barriers. These are also the two countries where it is most likely that education indeed increases access to material goods. The model has a poorer fit than the models in <Table 3 and are therefore not reported.

While the study offers unique data from sub-Saharan Africa, the four country samples are not representative at a country level and sampling was directed by bringing forth the uniqueness of each context, with variation in the rationale for selecting study sites. Any interpretation and use of results should take this into consideration.

The findings are in line with the results reported by Trani et al.[11,12] and van Rooy [8] regarding main barriers for access to health service. Coverage of health services in remote areas, highlighted by Trani et. al. as a main barrier was however not included in the current analyses. The four highest ranked barriers are perceived as either an insurmountable or very severe problem by more than one in five of the respondents. Transport costs, negative attitudes among health personnel, lack of accommodation at health facility, dangerous journey to facility, and accessibility to health facility are reported as insurmountable or very serious problems by clearly fewer than the main four barriers, but are also factors that need to be counted in as important barriers. While some barriers are perceived to be more important than others, all the barriers shown in Fig 2 require attention at health service level to improve access to health services in the four countries included in the study.

The study indicates that level of activity limitations (disability) is also associated with barriers, i.e., more severely disabled experience more problems. Socio-economic status comes out as the strongest predictor in the model, replicating findings from Sierra Leone [12]. This confirms that cost to accessing services is the major barrier. The association with level of education is substantially weaker, but indicates that education implies empowerment for individuals with disability.

It may be somewhat surprising that perceived barriers are higher in urban as compared to rural settings, as certain circumstances like terrain, distances, transport, and the availability of services generally are more problematic in many rural areas. This may be an effect of the complexity of urban contexts, substantial socioeconomic differences between population groups and even stronger effects of the relationship between disability and poverty as compared to rural contexts. The diversity, complexity and even price levels and/or domination of money economy may in fact increase the impact of disability on accessing services, as well as larger populations in urban areas competing for relatively limited services.

One aspect of the relationship between severity of disability and perceived barriers is that the association is clearly significant, but not very strong, and other predictors in the model seem to be even more important. When considering this result, one should bear in mind that in general, individuals with disability require more intervention from health services than non-disabled, and that services thus need to be particularly sensitive to the needs of vulnerable groups. This is particularly relevant when discussing equity in health. Several of the serious or insurmountable problems in accessing health care as shown in <Fig 2 deal directly with negative aspects of the service, including previous bad experiences, negative attitudes, refused access, etc. Even though the proportion of respondents indicating these aspects as serious/insurmountable problems is relatively small, these aspects indicate the opposite of what is required.

While most people face some problems in accessing health care in poor contexts, people with disabilities face additional and particular barriers, and those with greater disability face more barriers. To address these barriers requires an approach to health that stresses equity over equality; that is, addressing the specific barriers that exist for different types of service users. The provision of equitable health services can be supported by ensuring that evidence from research and practice contributes to policy revision and policy development which incorporates human rights and social inclusion as central features at international, regional and country levels [22].

## Supporting Information

S1 Data Data file used in the analyses. (XML)

S1 Syntax Syntax used for the analyses. (TXT)

## References

[1] MacLachlan M, Mannan H, MacAuliffe E. Access to health care of persons with disabilities as an indicator of equity in health systems. Open Med. 2011; 5: 1: e10–e12. PMC3205810 22046213

[2] London L. Issues of equity are also issues of rights: Lessons from experiences in South Africa, BMC Public Health. 2007; 14: 1–10. 10.1186/1471-2458-7-14 PMC1797007 17257421

[3] MacLachlan M, Swartz L (Eds.). Disability and international development: towards inclusive global health. New York: Springer: 2009.

[4] UN. Disability and the Millennium Development Goals A Review of the MDG Process and Strategies for Inclusion of Disability Issues in Millennium Development Goal Efforts. New York: United Nations: 2011.

[5] WHO. World Disability Report. Geneva: World Health Organization; 2011.

[6] WHO. Disability. Agenda item 13.5 at the Sixty-sixth World Health Assembly WHA66.9. Geneva: World Health Organization; 2013a.

[7] WHO. Report of the Technical Briefing. Preparing for the General Assembly High level Meeting on Disability and Development: The Health Sector's Contribution 66th World Health Assembly, 23rd 5 2013 Geneva: World Health Organization; 2013b.

[8] Van Rooy G, Amadhilaa E M, Mufuneb P, Swartz L, Mannan H, MachLachlan M. Perceived barriers to accessing health services among people with disabilities in rural northern Namibia. Disability and Society. 2009; 10.1080/09687599.2012.686877

[9] Tomlinson M, Swartz L, Officer A, Chan K Y, Rudan I, Saxena S. Research priorities for health of people with disabilities: an expert opinion exercise. Lancet. 2009; 374: 1857–62. 10.1016/S0140-6736(09)61910-3 19944866

[10] WHO. World Health Survey. Geneva; World Health Organization: 2010.

[11] Trani J-F, Bakhshi P, Noorb A A, Lopez D, Mashkoor A. Poverty, vulnerability, and provision of healthcare in Afghanistan. Social Science & Medicine. 2010; 70: 1745–1755. 20359809 10.1016/j.socscimed.2010.02.007

[12] Trani J-F, Browne J, Kett M, Bah O, Morlai T, Bailey N, Groce N. Access to health care, reproductive health and disability: A large scale survey in Sierra Leone. Social Science & Medicine. 2010; 70: 1745–1755 2011, 73, 1477–1489. 22014873 10.1016/j.socscimed.2011.08.040

[13] Washington Group on Disability Statistics. Understanding and interpreting disability as measured using the WG Short Set of Questions. Washington Group on Disability Statistics (WG), 2009; [http://www.cdc.gov/nchs/data/washington_group/meeting8/interpreting_disability.pdf], 23^rd^ October 2014.

[14] Eide A H, Jele B. Living Conditions among People with Disabilities in Swaziland. A National, Representative Study SINTEF A 20047. Oslo; SINTEF Technology & Society: 2011.

[15] Kline R B. Principles and Practice of Structural Equation Modeling. 2nd ed Guilford Press, New York, 2005.

[16] UNDP. Human Development Report 2014 Sustaining Human Progress: Reducing Vulnerabilities and Building Resilience. New York; United Nations Development Programme: 2014.

[17] Madans J H, Loeb M E, Altman B M. Measuring disability and monitoring the UN Convention on the Rights of Persons with Disabilities: the work of the Washington Group on Disability Statistics. BMC Public Health. 2011; 11 (Suppl 4): S4 http://www.biomedcentral.com/content/pdf/1471-2458-11-S4-S4.pdf 10.1186/1471-2458-11-S4-S4 21624190 PMC3104217

[18] United Nations Statistical Commission. Report of the Washington Group on Disability Statistics. Report no: E/CN.3/2007/4. Geneva; United Nations Economic and Social Council: 2007.

[19] Hooper D, Coughlan J, Mullen M. Structural equation modelling: guidelines for determining model fit. Electronic Journal of Business Research Methods. 2008; 6: 1: 53–60.

[20] Mueller R O, Hancock G R. Best practices in structural equation modeling In: Osborne J W (ed). Best Practices in Quantitative Methods. 2008; SAGE: Thousand Oaks, CA: p. 488–510.

[21] Schreiber J B. Core reporting practices in structural equation modeling. Research in Social and Administrative Pharmacy. 2008; 4: 2:83–97. 10.1016/j.sapharm.2007.04.003 18555963

[22] Amin M, MacLachlan M, Mannan H, El Tayeb S, El Khatim A, Swartz L, Munthalim A., van Rooy G, McVeigh J, Eide A., Schneider M. EquiFrame: A framework for analysis of the inclusion of human rights and vulnerable groups in health policies. Health & Human Rights. 2011; 13: 2: 1–20. http://www.hhrjournal.org/index.php/hhr/article/view/430/694 22957368

